# Attribution of hydrological change in Heihe River Basin to climate and land use change in the past three decades

**DOI:** 10.1038/srep33704

**Published:** 2016-09-20

**Authors:** Kaisheng Luo, Fulu Tao, Juana P. Moiwo, Dengpan Xiao

**Affiliations:** 1 Key Laboratory of Land Surface Pattern and Simulation, Institute of Geographic Sciences and Natural Resources Research, Chinese Academy of Sciences, Beijing 100101, China; 2 University of Chinese Academy of Sciences, Beijing 100101, China; 3 Natural Resources Institute Finland (Luke), 01301 Vantaa, Finland; 4 Department of Agricultural Engineering, School of Technology, Njala University, Sierra Leone; 5 Institute of Geographical Sciences, Hebei Academy of Sciences, Shijiazhuang 050011, China

## Abstract

The contributions of climate and land use change (LUCC) to hydrological change in Heihe River Basin (HRB), Northwest China were quantified using detailed climatic, land use and hydrological data, along with the process-based SWAT (Soil and Water Assessment Tool) hydrological model. The results showed that for the 1980s, the changes in the basin hydrological change were due more to LUCC (74.5%) than to climate change (21.3%). While LUCC accounted for 60.7% of the changes in the basin hydrological change in the 1990s, climate change explained 57.3% of that change. For the 2000s, climate change contributed 57.7% to hydrological change in the HRB and LUCC contributed to the remaining 42.0%. Spatially, climate had the largest effect on the hydrology in the upstream region of HRB, contributing 55.8%, 61.0% and 92.7% in the 1980s, 1990s and 2000s, respectively. LUCC had the largest effect on the hydrology in the middle-stream region of HRB, contributing 92.3%, 79.4% and 92.8% in the 1980s, 1990s and 2000s, respectively. Interestingly, the contribution of LUCC to hydrological change in the upstream, middle-stream and downstream regions and the entire HRB declined continually over the past 30 years. This was the complete reverse (a sharp increase) of the contribution of climate change to hydrological change in HRB.

Water is increasingly a limiting factor to socioeconomic development, especially in arid and semi-arid regions^1^. Water scarcity can endanger food security and sustainable economic development, as well as the health of the ecosystem^2^,^3^. Hence changes in water quantity have become the focus of attention in river basin management and ecological restoration.

Addressing the issue of water shortage requires knowledge of the factors which drive hydrological changes and the related effects on local water resources. The effects of climate and land use change (LUCC) on water resources have attracted much attention over the years^4^,^5^. In terms of global warming and extreme weather frequency and intensify^6^,^7^, climate change has been identified for the reductions in global water resources^8^,^9^ and exacerbation of water shortage in arid and semiarid regions. Also variations in regional hydrological cycles are closely related with LUCC^10^,^11^,^12^. Temperature increases with increasing global warming, causing changes in precipitation pattern and intensity which in turn significantly affect regional hydrological cycle^13^.

Documented literature reveals that often, several hydrological studies only deal with specific components of water balance, e.g., streamflow^14^,^15^,^16^, groundwater recharge^17^,^18^,^19^,^20^, runoff 21,22,23,24 and evapotranspiration^25^,^26^,^27^,^28^. Land use affects stream runoff 29,30,31, water infiltration capacity^32^,^33^ and surface evaporation^34^,^35^,^36^. However, few studies have focused on the evaluation of basin water balance in terms of the impact of LUCC and climate change on hydrological processes.

Reviews of several domestic and international studies suggest that LUCC and climate change have significant effects on the hydrological components of a river basin^37^,^38^. However, it remains unclear which of the two factors (LUCC and climate change) dominantly contribute to basin hydrological processes. There is also a need to know the temporal and spatial changes in the contributions of LUCC and climate change to basin hydrological change. Thus this study quantified the contributions of LUCC and climate change to a typical basin hydrological change in Northwest China temporally and spatially, which will deepen the existing understanding about the interaction of the selected hydrological factors and the implications for water resources management.

Heihe River Basin (HRB), the second largest inland river basin in China, was investigated in this study (see Supplementary Fig. S1). Because of the fragile ecological environment and severe water scarcity^37^, effective management is crucial for HRB hydrology. Water scarcity in the basin has caused significant changes in the local hydrological environment over the past decades; including environmental degradation, salinization and desertification^37^. The change in hydrological regimes induced by LUCC and climate change^37^ is less understood in the basin.

The main objectives of this study were: 1) to quantify the contributions of LUCC and climate change to hydrological processes in HRB in Northwest China; and 2) to determine the temporal and spatial trends in the contributions of LUCC and climate change to the basin hydrological change. To do this, SWAT (Soil and Water Assessment Tool) model was adapted to HRB using climate, hydrology, soil, land use and DEM data, and run for 11 scenario experimental conditions. To estimate the contributions of LUCC and climate change to the basin hydrological change, one factor was changed at a time with the others held constant. Specifically, 11 model scenarios (i.e., M1, M2, M3, …, M11) were used to quantify the contributions of LUCC and climate change to the basin hydrological change. The Man-Kendall trend test was used to analyze the dynamics of temperature, precipitation and water yield in the basin (see Methods).

## Results

### Model calibration and validation

Based on the distribution of the natural drainage network, basin topography and rainfall, the HRB was delineated into 589 sub-basins. The sub-basins were further divided into 6850 Hydrologic Response Unit (HURs) based on the basin land use, soil property and slope. The Nash-Sutcliffe efficiency coefficient (*NS*), *R*^2^ coefficient, percent bias (*PBIAS*) and RMSE-observation standard deviation ratio (*RSR)* were used to assess the reliability and accuracy of the model simulation. The accuracy measures at the yearly scale were 0.65 < *NS* < 0.75, 0.50 < *RSR* < 0.60, and 10% < *PBIAS* < 15% for both the calibration and validation periods. This suggested that the model performance was fairly good (Table S1), especially for the upstream region (UHRB), although the performance at monthly scale was not as good as at yearly scale (Table S2). The actual model performance was best for the downstream region (DHRB). This was because the observed river discharge was either small or dried up, making the comparison impossible with the simulated values. The measured water yield was further compared with the simulated water yield at decadal scale. The comparison showed that the SWAT model performed fairly well, with a relative error range of 2.02‒3.42% (Table S3).

### Land use change (LUCC) and climate change in 1980‒2000

Compared with the middle-stream (MHRB) and downstream (DHRB) regions, LUCC was smallest in the upstream (UHRB) region in 1980‒2009 (see Supplementary Fig. S2). With the exception of the 1990s, agricultural land steadily expanded while forest land shrunk.

In the UHRB, the area of forest decreased in the 1980s, 2000s and during 1980‒2009, but increased in the 1990s (see Supplementary Fig. S2). Pasture land decreased in the 1980s, but increased in the 1990s and the 2000s in the UHRB. The variation in land use was largest in the MHRB during 1980‒2009, with the largest change in the 1990s. As shown in Fig. S2 (see Supplementary), the largest fluctuation was for agricultural land. This was verified by published data in the statistics yearbook. Bare land (including desert and the Gobi land) expanded in the 1980s in the DHRB, but decreased since the early 1990s. Agricultural land steadily expanded since the 1980s in the study area.

Annual mean temperature increased significantly in the UHRB, MHRB, DHRB and the entire HRB during 1980‒2009 (Figure S3, Supplementary). The largest change rate (0.62 °C/10a) was in the DHRB, followed by the UHRB (0.56 °C/10a) and then the MHRB (0.52 °C/10a). Annual precipitation increased slightly in the entire HRB (7.77 mm/10a) and in the three sub-regions, but none of the increases was significant (*p* > 0.05). The maximum increase was in the UHRB (12.21 mm/10a), followed by the MHRB (5.90 mm/10 yr) and then the DHRB (5.71 mm/10 yr) (see Supplementary Fig. S3).

### Change in hydrological processes

#### Water yield change

**Figure 1:**
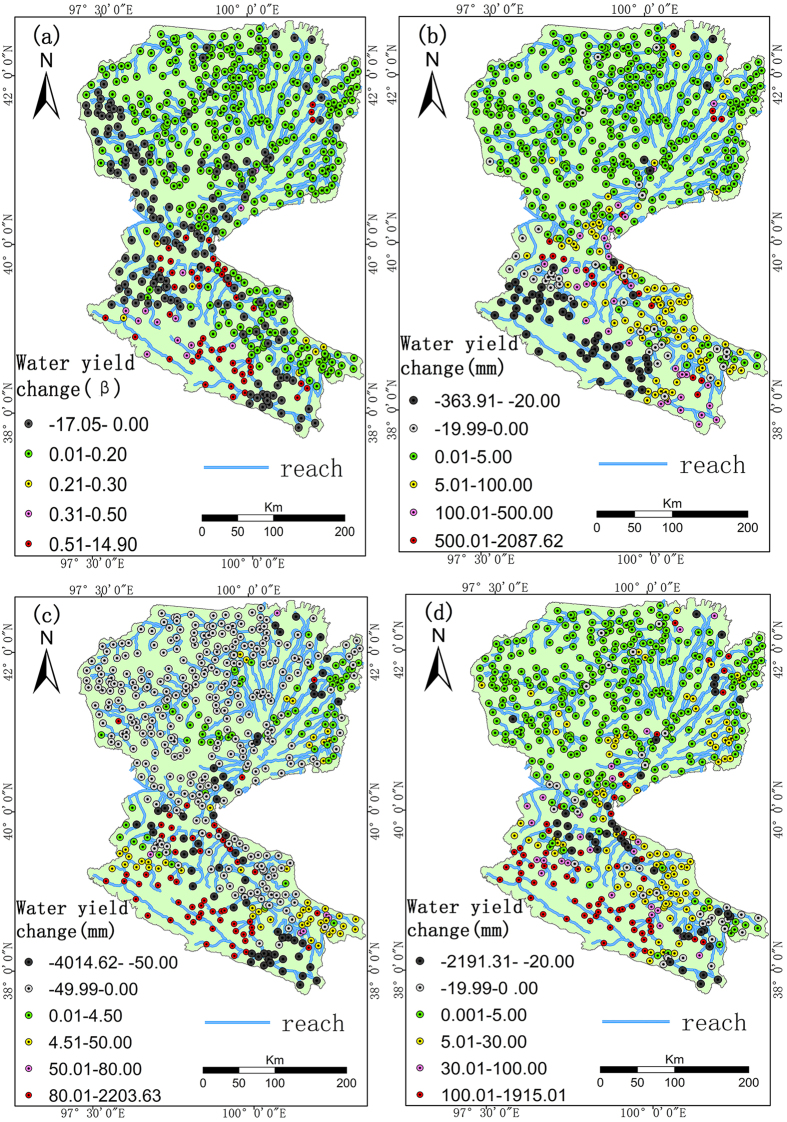
Plots of β value for 1980‒2009 (**a**), change in water yield in the 1980s (**b**), 1990s (**c**) and 2000s (**d**) in Heihe River Basin (HRB) in Northwest China. This figure was generated though the ArcGIS 10.2 software provided Environmental Systems Research Institute (http://www.esri.com).

Figure 1a depicts the changes in water yield and the related spatial patterns during 1980‒2009. Overall, the increase in water yield was most obvious in the UHRB and MHRB during 1980‒2009. However, there were clear differences in the spatial patterns, which was most obvious for the UHRB (0.3‒14.9 mm), followed by the MHRB and then the DHRB. Water yield generally increased in the entire HRB in the 1980s, with exception of the UHRB for which it was −363‒20 mm (Fig. 1b). There was an overall decline in water yield in the entire HRB in the 1990s, except for the UHRB (Fig. 1c). The increase in water yield in most of the sub-basins in the UHRB exceeded 80 mm in the 2000s (Fig. 1c).

#### Water balance

**Figure 2:**
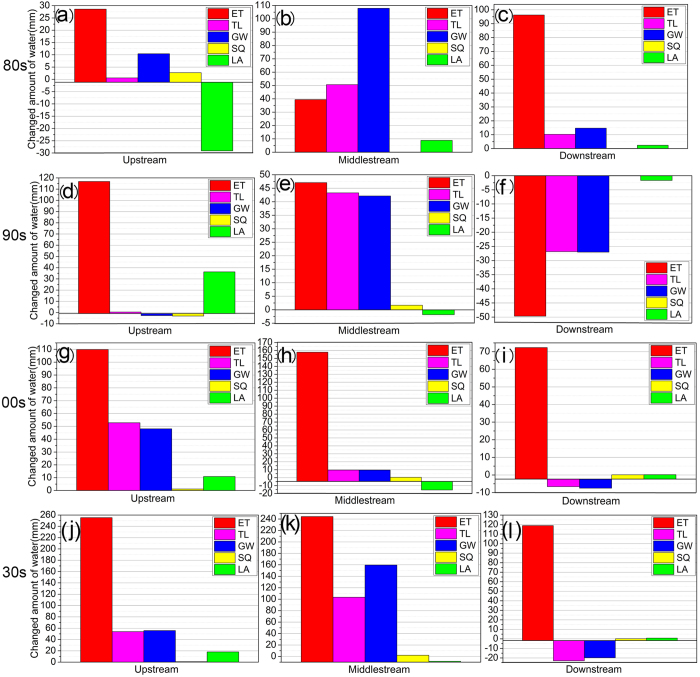
Plots of the combined effects of land use and climate change on the hydrology Heihe River Basin (HRB) in the 1980s (**a**–**c**), 1990s (**d**–**f**), 2000s (**g**–**i**) and 1980‒2009 (**j**–**l**). ET is actual evapotranspiration; SQ is surface runoff into reach (mm); GW is base-flow into reach (mm); TL is transmission loss; and LA is lateral flow contribution to stream-flow (mm).

The changes in water balance mainly reflected the combined effects of LUCC and climate change in the basin. Figure 2 depicts the changes in actual evapotranspiration (ET), surface runoff (SQ), groundwater recharge (GW), lateral flow (LA) and transmission loss (TL), both positive and negative trends existed for 1980‒2009 in the basin. Under the combined effects of land use and climate change, the largest increase in ET in the UHRB was in the 2000s (157.78 mm), followed by the 1990s and then the 1980s. There was also an increasing effect of LUCC and climate change on ET in the MHRB. However, because of the small SQ, the change in SQ was less than 2.5 mm in both the MHRB and DHRB.

GW increased in the UHRB and MHRB, with the largest recharge in the 2000s in the UHRB and in the 1980s in the MHRB, however the trend weakened with time. GW in the DHRB had decreased since the 1990s, with the largest decline in the first decade. The trend in LA was positive for the UHRB in the 1990s and the 2000s, for the MHRB in the 1980s and for the DHRB in the 1980s and 2000s. Also, it was positive for the UHR in the 1980s, for the MHRB in the 1990s and 2000s, and for the DHRB in the 1980s (Fig. 2). Overall, the change in ET due to the combined effect of LUCC and climate change in the three regions was largest during 1980‒2009, and that in SQ was largest for the MHRB.

**Figure 3:**
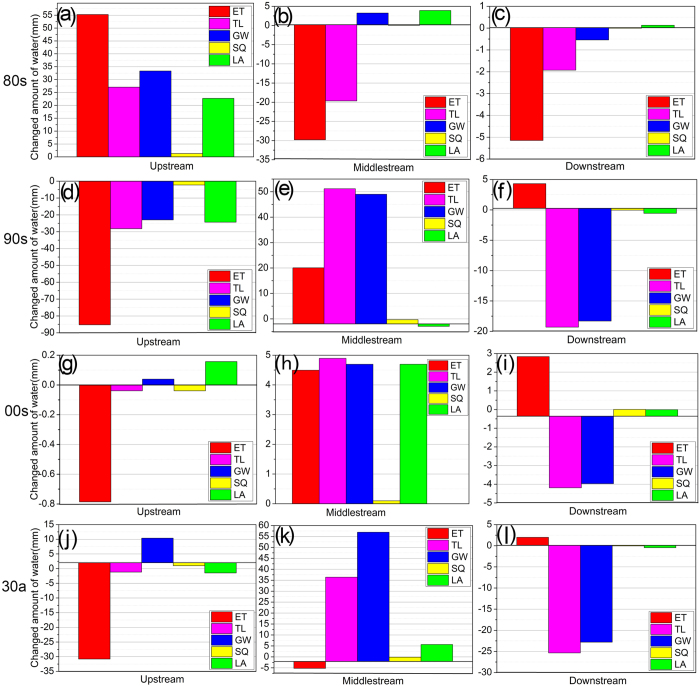
Plots of the effects of land use on the hydrology of the Heihe River Basin (HRB) in the 1980s (**a**–**c**), 1990s (**d**–**f**), 2000s (**g**–**i**) and 1980‒2009 (**j**–**l**). ET is actual evapotranspiration; SQ is surface runoff into reach (mm); GW is base-flow into reach (mm); TL is transmission loss; and LA is lateral flow contribution to stream-flow (mm).

The contribution of LUCC to the water balance is shown in Fig. 3. The maximum change in ET (−85.27 mm) was in the 1990s for the UHRB. Then it had the largest effect in the UHRB, followed by the MHRB and DHRB. The contribution of LUCC to ET change increased in 1980‒2009 for the MHRB and DHRB. The effect of LUCC on SQ was relatively small in the MHRB and DHRB. The largest effect of land use on GW and TL was in 1980‒2009 for the MHRB, followed by the UHRB and DHRB. Although the contribution of LUCC to the upstream hydrological change generally decreased, it had the largest impact on LA for UHRB, followed by the MHRB and DHRB. This was closely related to the transition trend in LUCC, especially with decreasing forest and pasture lands from the upstream to the downstream regions.

**Figure 4:**
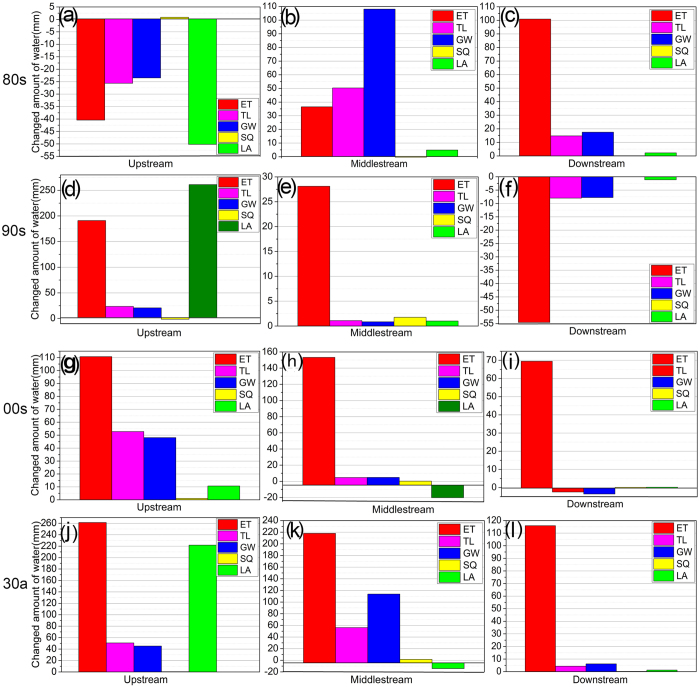
Plots of the effects of climate change on the hydrology Heihe River Basin (HRB) in the 1980s (**a**–**c**), 1990s (**d**–**f**), 2000s (**g**–**i**) and 1980‒2009 (**j**–**l**). ET is actual evapotranspiration; SQ is surface runoff into reach (mm); GW is base-flow into reach (mm); TL is transmission loss; and LA is lateral flow contribution to stream-flow (mm).

Figure 4 shows the contribution of climate change alone on the entire water balance in HRB. Due to climate change, ET increased in 1980‒2009 for the HRB, in the 1980s for the UHRB and in the 1990s for the MHRB. During 1980‒2009, the increase in ET was 261.35 mm for the UHRB, followed by the MHRB (217.94 mm) and DHRB (115.88 mm). Overall, SQ increased slightly during 1980‒2009 in the HRB. GW increased in the HRB during 1980‒2009 due to climate change, but the trend was negative for the 1980s in the UHRB and for the 1990s and 2000s in the MHRB. The effect of climate change was most obvious in the UHRB. In the DHRB, climate change mainly affected ET, which increased by 115.88 mm during 1980‒2009.

### Contribution of LUCC and climate change to HRB hydrological change

**Figure 5:**
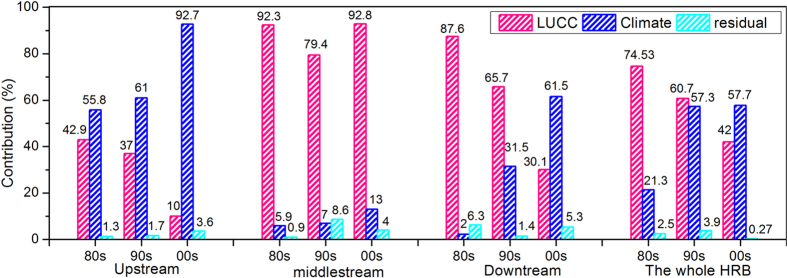
The contributions of land use and climate change to the entire hydrology of the Heihe River Basin (HRB).

The contribution of LUCC to the basin hydrological change was largest (92.3%) in the MHRB (Fig. 5). For climate change, the contribution was largest (55.8%) in the UHRB and smallest (2.0%) in the DHRB. In the UHRB, the contribution of climate change to the hydrological change was 12.9% higher than that of land use in the 1980s. Thus during this period, the basin hydrological change was mainly driven by climate change. However, LUCC was the driving factor of the basin hydrological change in the MHRB and DHRB. The contribution of LUCC to the hydrological change in the 1980s was 86.4% higher than that of climate change in the MHRB and 85.6% higher than that of climate change in the DHRB. Due to uncertainty in the model simulation, residual effect was largest in the DHRB. This showed that in the 1980s the UHRB hydrological change was driven more by climate change whereas that in the MHRB and DHRB was driven more by LUCC. For the hydrological change in the whole HRB, the contribution of LUCC was 53. 2% higher than that of climate change.

In the 1990s, contribution of LUCC to hydrological change was largest (79.4%) in the MHRB and smallest (37.0%) in the UHRB. And contribution of climate change to hydrological change was largest (61.0%) in the UHRB and smallest (7.0%) in the MHRB. The hydrological change in the UHRB was mainly driven by climate change, accounting for 24% more than LUCC in the 1990s. Then the hydrological change in the MHRB and DHRB was mainly driven by land use, accounting for 72.4% and 34.2% more than climate change, respectively. In the entire HRB, the contribution of LUCC to hydrological change was 3.4% greater than that of climate change.

LUCC was the main driving factor in the 2000s, accounting for 92.8% of the hydrological change in the MHRB. This agreed with the increase in contribution of climate change in the 2000s, with peak levels of 92.7% and 61.5% in the UHRB and DHRB, respectively (Fig. 5). The contributions of land use and climate change to the hydrological change in the entire HRB were 42.0% and 57.7%, respectively. This suggested that the effect of climate change gradually surpassed that of LUCC and became the main factor influencing the hydrological change in the HRB in Northwest China.

## Discussions

### LUCC and basin hydrological change

It was expected that changes in land use will continue in the future, which will have significant effect on regional water balance. Human disturbance of land cover systems has significantly altered the Earth’s land surface^39^, substantively reshaping global water balance^40^. The intensification of land use, population growth and socio-economic development continues to exert a huge pressure on available water resources^40^.

Deforestation decreases soil water retention capacity, and increases SQ by accelerating water movement due to low resistance to flow and low soil compaction^41^. In contrast, reforestation increases water retention capacity, and thereby decreases SQ by decelerating water movement due to high resistance to flow and longer flow paths^42^. Runoff was largest in the UHRB, which is the most forested sub-basin in the HRB. Forest area decreased in the 1980s, 2000s and in 1980‒2009, but increased in the 1990s. These changes, resulting in decreasing albedo and increasing SQ in the 1980s‒2000s, led to the decline in ET. Nevertheless, the changes in land surfaces, groundwater and pasture somehow compensated the effects of deforestation on the basin water resources.

Surface roughness, albedo and other properties that affect the exchange of water and energy between the land surface and the atmosphere are altered by LUCC. This results in the variability of surface energy and net radiation^39^, which in turn influences hydrological processes. The expansion of agricultural lands has a significant effect on shallow aquifer water system due to the associated increase in irrigation^43^. Over 80% of irrigated agricultural lands in the HRB are located in the MHRB. In the past 30 years, agricultural land increased by 579.4 km^2^, and most of which (499.7 km^2^) was in the MHRB. Change in agricultural land was the main form of LUCC in the middle-stream region, with a major impact on water balance. With the expansion of agricultural land in the 1990s and 2000s, TL and GW increased (due to enhanced infiltration) and ET increased (due to flood irrigation). In the 1980s, TL and ET decreased with the shrinking of agricultural lands. On the average, agricultural lands expanded steadily in the Ejnaqi Oasis by 86.9 km^2^ in 1980‒2009. Also because of this, the oasis rapidly degraded into desert, which in turn affected water balance in the DHRB.

Irrespective of the seasons in a year, degradation of productive lands into barren lands induces the most change in total heat flux. In the HRB, ET was mainly driven by available energy. The conversion of grassland into barren or sparsely vegetated lands limited available energy for ET, which in turn accelerated runoff 34. Desert and oasis are the dominant landscape in HRB. Desert is dominant in the MHRB and DHRB, accounting for 73.0% in the DHRB. Desertification was therefore the main mode by which LUCC influenced water balance in the study area. The expansion of deserts in the past 30 years was 61.9 km^2^, significantly influencing water availability in the DHRB. There was a clear negative correlation between change in ET and time during 1980‒2000. Also for 1980‒2009, desertification reduced TL and GW, resulting in low SQ and LA in the DHRB.

The contribution of LUCC to the basin hydrological change was 87.4% in the 1980s, which dropped to 30.1% in the 2000s. The average contributions of LUCC to upstream, middle-stream and downstream hydrological change in 1980‒2009 were 30.3%, 88.2% and 61.1%, respectively. Thus unlike the UHRB, LUCC dominantly influenced hydrological change in the MHRB and DHRB in 1980–2009. Forest and pasture accounted for over 90% in the UHRB, which, along with sparse population, mitigated the effect of human activity on the basin hydrological change. The MHRB is a predominantly agricultural-based basin with a relatively dense population, hence the Gansu side of the Hexi Corridor is a main grain production region^44^, and human activity has a major impact on the local hydrological change.

The results are supported by several previous studies^45^,^46^,^47^. Compared with climate change, human activity has larger effect on the hydrological processes in the MHRB^45^. Due to human activity, the heterogeneity and diversity of the landscape have been deteriorating since the early 1980s^37^. Human activity was the main driver of desertification in MHRB in 1860‒1999^37^,^45^, particularly as driven by the temporal and spatial pattern of water resources in the region^46^. Vegetation degradation in DHRB was mainly driven by intensive human activity in the region in the past five decades^47^.

### Contribution of climate change to hydrological change in the entire basin

Annual mean temperature in the HRB increased with increasing global warming, the rate was much higher (0.054 °C/yr) than the global mean (0.012 °C/a)^48^,^49^, and especially in the DHRB (0.062 °C/yr). Warming climatic conditions are expected to increase evaporation rate^50^. Although overall ET increased notably in the HRB in 1980‒2009, this was more obvious in the UHRB where it had the largest effect due to scarce water resources. Obviously, the changes in ET were due to climate change^51^. Low ET in the 1980s in the UHRB and in the 1990s in the DHRB was due to low wind speed, which reduced the role of rising temperature in the basin hydrological change.

There was a weak increase in annual precipitation, resulting in an overall increase in the basin SQ in 1980‒2009. The largest increase in SQ was in the MHRB, followed by the UHRB and DHRB. In the UHRB, SQ increased due to increased summer precipitation and warming winter conditions^52^. The widespread of forest in the region enhanced soil water retention and thereby decreased SQ. Currently, in the MHRB, there are over 80% agriculture land and 86% impervious surface, which significantly increase SQ. Because of high ET (driven by high temperature and the vast desert and the Gobi), SQ was very small in the DHRB. Climate change had the largest effect on GW, LA and TL, and thus on ET and SQ, with the largest effect in the UHRB.

ET was the main factor influencing the contribution of climate change to the hydrological change in MHRB and DHRB. The contribution of climate change to the hydrological change in the upstream basin was 55.8% (1980s), 61.0% (1990s) and 92.7% (2000s). The contribution of climate change to the hydrological change in middle-stream basin was 5.9% (1980s), 7.0% (1990s) and 13.0% (2000s). It was 2.0% (1980s), 31.5% (1990s) and 61.5% (2000s) in the downstream basin. Thus the average contributions of climate change to the hydrological change in the upper, middle and down steam basins in 1980‒2009 were 21.3%, 57.3% and 57.7%, respectively. Although it was only 36.7% for the whole HRB in 1980‒2009, it obviously increased with climate warming. SQ and ET increased with increasing temperature and precipitation^53^, further influenced runoff and hydrological processes in the MHRB and HRB^54^,^55^.

### Temporal and spatial changes in analyzed contibutions

There was a large spatial variation in the contributions of LUCC and climate change to the hydrological change in the HRB. In the past three decades, the effect of climate change on the basin hydrological change was largest for UHRB, followed by DHRB and then MHRB. The largest contribution of LUCC to hydrological change was in the MHRB, followed by the DHRB and UHRB. There is little human activity in the study area because of the sparse population and harsh natural environment^51^. The MHRB has the largest population and the most robust economy in the HRB in Northwest China^44^,^45^. Interestingly, the contribution of LUCC to hydrological change continually decreased in the upstream, middle-stream, downstream basins and then in the entire HRB over the past 30 years. On the contrary, the contributions of climate change to the hydrological change in the basin sharply increased. The trends could continue in the future due to the projected climate change^56^,^57^.

### Adaptive strategies and options

The analysis on the attribution of the basin hydrological changes to LUCC and/or climate change is fundamental to basin water resources management and planning. This study indicated that the contributions of LUCC and climate change to basin hydrological change varied with regions due to the heterogeneity of hydrological conditions^58^. This suggested that the level of priority on LUCC management and climate change adaptation should be made for different regions. For instance, the forest region of the UHRB (as the water source region of HRB) should be strictly protected because of its critical role in protecting hydrologic function. In the MHRB, it is urgent to control the rate and scale of expansion of agricultural lands because of its negative effect on hydrological cycle. Water consumption in industrial and domestic sectors is small in the HRB, but a huge 87% of total water use is from the agricultural sector^37^. Increasing agricultural water use with increasing population in the MHRB has occupied ecological water use in the DHRB^37^. The current flood irrigation is the least efficient way of agricultural water use, which results in a considerable waste of the limited available water resources in the basin^43^,^44^. To address the water shortage problem, some less productive croplands should be transformed into grassland and some high water consumption cropping systems should be transformed into low water consumption ones. Efficient water allocation should be emphasized for ecological, agricultural, industrial and human consumption.

## Methods

### Site description

The HRB lies in Northwest China, within 38‒42° N and 98‒101° W, and covers an area of 14.31 × 10^5 ^km^2^ (see Supplementary Fig. S1). The basin is divided into three sub-basins — the upstream basin (UHRB) of the Qilian Mountains in Qinghai Province; the middle-stream basin (MHRB) of the oases and irrigated lands in Gansu Province, and the downstream basin (DHRB) of the Gobi Desert in Inner Mongolia^53^. There are 17 main tributaries (41 perennial tributaries) in the basin originating from Qilian Mountains. The estimated mean runoff from the tributaries is 34. 43 × 10^8 ^m^3^/yr. Heihe River (the main river in HRB) is the largest perennial river, with a total length of 812 km and runoff of 15.8 × 10^8 ^m^3^/yr, accounting for half of the total runoff in the basin. As a hinterland river basin in continental Asia, HRB has an arid continental monsoon climate that is extremely hot in summer and severely cold in winter, with 60–70% of the precipitation occurring during July to September^37^.

### Hydrological model setup

The Soil and Water Assessment Tool (SWAT), which is a physically-based, semi-distributed hydrological model was used in this study. Because SWAT is a deterministic model, each successive model run that uses the same inputs gives the same outputs. This type of model is suitable for isolating the effects of a single variable, allowing the impact of change to be isolated and quantified relative to the contributions of other variables. Because of this, the SWAT model and its variants are widely used around the world^58^,^59^. A comprehensive index of water yield (*Y*_*water*_) was used to characterize the total effects on hydrology in the investigated basin. *Y*_*water*_ = SQ + LA − TL − Pond: where *SQ* is surface runoff into reach (mm); *GW* is baseflow into reach (mm); *TL* is transmission loss [mm]; *LA* is lateral flow contribution to streamflow (mm); and Pond is intercepted water from reservoirs/ponds (mm). Reservoir/pond interception was negligible in this study because the limited number/area of reservoirs/ponds in the basin. The contributions of LUCC and climate change to hydrological changes in the basin were quantified by comparison with different climate scenarios.

The model was pre-run for the period 1975‒1981 to stabilize the used parameters. Then data for 1981‒1997 were used for calibration and 1998‒2010 for validation. The Nash-Sutcliffe efficiency coefficient (*NS*) and coefficient of determination (*R*^2^) were used to evaluate the model performance, comparing simulated and observed discharge at both monthly and yearly scales. The Nash-Sutcliffe efficiency coefficient (*NS*), *R*^2^ coefficient^59^, percent bias (*PBIAS*) and RMSE-observation standard deviation ratio (*RSR)*^60^,^61^ were used to assess the reliability and accuracy of the model simulation. Generally, a model simulation is rated as good if 0.65 < *NS* < 0.75, 0.50 < *RSR* < 0.60 and ±10% < *PBIAS* < ±15%. A model simulation is judged as satisfactory if 0.50 < *NS* < 0.65, 0.60 < RSR < 0.70 and ±15% < *PBIAS* < ±25%^60^.

With snow hydrology module, SWAT is also suitable for simulating snowmelt runoff 61,62. In SWAT, the areal coverage of snow in a basin is defined using areal depletion curve, which describes seasonal growth and recession of snowpack as a function of the amount of snow in the sub-basin. Snowmelt is controlled by air and snowpack temperature, melting rate and areal snow coverage. Three parameters control snowfall accumulation — snowfall temperature threshold (SFTMP), areal snow coverage threshold at 100% (SNOCOVMX) and areal snow coverage threshold at 50% (SNO50COV). Another four parameters govern SWAT snowmelt estimation — snowpack temperature lag (TIMP), snowmelt base temperature (SMTMP), maximum melt factor (SMFMX) and minimum melt factor (SMFMN)^58^. To reduce uncertainty, a suitable parameter was selected through sensitivity analysis using the Latin Hypercube approach at the 0.001 step length. Agricultural areas can be irrigated using diversions from within the sub-basin or outside the sub-basin. All irrigation parameters adopted in the model were the model default values.

### Data

The soil data used in the SWAT simulation were from Harmonized World Soil Database (HWSD). The land use data (including 1985, 1995, 2000, 2005 and 2008) were from Environmental Data Center of Chinese Academy of Sciences. Then land use data for 2005 were used to build the model and the others used to construct scenario conditions. The land use status in each decade was represented using the mid-year land use maps for the HRB. The land use maps for 1985, 1995 and 2005 were considered to be sufficiently representative of land use in the 1980s.1990s and 2000s, respectively. Weather data for the SWAT model simulation were obtained from China Meteorological Administration. Daily climate data for 1980‒2012 were from 14 weather stations administrated by China Bureau of Meteorology. Hydrological data were from Heihe River Water Protection Bureau and China Hydro-Statistical Yearbook. Monthly mean discharge data used for the model calibration and validation were from 11 gauge stations in the basin. Shuttle Rader Topography Mission Digital Elevation Model (DEM) with a resolution of 30 m was obtained from West Data Center of China (WDCC).

### Analysis

To estimate the contributions of LUCC and climate change to the hydrological change in the HRB, one factor was changed at a time and the rest held constant. Specifically, 11 model scenarios (i.e., M1, M2, M3, …, M11) were run to quantify the contributions of LUCC or climate change to the basin hydrological change. The M01 to M04 scenarios were used to quantify the contributions of LUCC and climate change to HRB basin hydrological change in the 1980s. Then scenarios M4 to M7 were used to quantify the contributions of LUCC and climate change to hydrological change in the 1990s. Also, scenarios M8 to M11 were used for the contributions of LUCC and climate change to hydrological change in the HRB in the 2000s (Table 1). The contribution for each decade was evaluated by comparing the differences of simulated water yield between two scenarios in the HRB and three sub-basins (Table 2).

A representative land use for the 1980s and climate data for 1980‒1989 (hereafter called 1980s climate) were denoted by modeling scenario M1. Then a representative land use for the 1990s and climate data for 1990‒1999 (hereafter called 1990s climate) were denoted by modeling scenario M4. The hydrological change in 1980s due to the combined effects of LUCC and climate change was calculated as the difference between M4 and M1 (Y_M4_-Y_M1_). A representative land use for the 1980s and climate data for 1990‒1989 (hereafter called 1990s climate) were denoted by modeling scenario M2. Also a representative land use for 1990s and climate data for 1980‒1989 (hereafter called 1980s climate) were denoted by modeling scenario M3. The contribution of climate change to hydrological change in the 1980s was then calculated as:


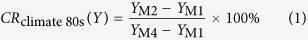


where *CR*_climate 80s_ is the contribution of climate change to hydrological change in the 1980s; *Y*_M4_, *Y*_M2_ and *Y*_M1_ are the water yields of model scenarios M4, M2 and M1, respectively.

Similarly, the contribution of LUCC to hydrological change in the 1980s was quantified by comparing model scenarios M1, M2 and M4. Hence, the contribution of LUCC to hydrological change in the 1980s was calculated as:


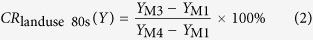


where *CR*_landuse 80s_ is the contribution of land use to hydrological change in the 1980s; *Y*_M1_, *Y*_M3_ and *Y*_M4_ are the water yields of model scenarios M1, M3 and M4, respectively.

(Y_M4_ − Y_M1_) − (Y_M2_ − Y_M1_) − (Y_M3_ − Y_M1_) was the residual error of the model simulations (Table 3). Residual error is a model induced error calculated as:


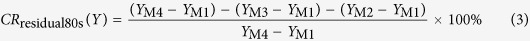


where *CR*_residual 80s_ is the contribution of residual error to the calculations for the 1980s; and the other variables defined as above.

A similar method was used to calculate the contribution of LUCC and climate change to hydrological change in the UHRB, MHRB and DHRB sub-basins and then the entire HRB in the 1990s and 2000s.

Linear regression analysis was used to depict the trends in precipitation and temperature in the entire basin study area and then the three sub-basins. Using the Mann-Kendall trend analysis, annual water yield (*β*) was calculated for each of the 589 third-tier sub-basins and then summed up for the three second-tier sub-basins of the HRB. A positive *β* suggests an increasing trend and vice versa. Then the larger the absolute value of *β*, the faster and larger the trend of change.

## Additional Information

**How to cite this article**: Luo, K. *et al*. Attribution of hydrological change in Heihe River Basin to climate and land use change in the past three decades. *Sci. Rep*. **6**, 33704; doi: 10.1038/srep33704 (2016).

## Supplementary Material

Supplementary Information

## Figures and Tables

**Table 1:** A list of the different model scenarios used to evaluate the impacts of land use and climate change on the hydrology of Heihe River Basin (HRB) in Northwest China.

Scenario	Land use	Climate change
M1	1980s	1980s
M2	1980s	1990s
M3	1990s	1980s
M4	1990s	1990s
M5	1990s	2000s
M6	2000s	1990s
M7	2000s	2000s
M8	2000	2000‒2006
M9	2000	2007‒2013
M10	2008	2000‒2006
M11	2008	2007‒2013

Note that land use maps for 1985, 1995 and 2005 were used to represent land use in the 1980s, 1990s, 2000s, respectively; climatic condition of the 1980s is denoted by climatic data for 1980–1989; climatic condition of the 1990s is denoted by climatic data for 1990–1999; and then climatic condition of the 2000s is denoted by climatic data for 2000–2009.

**Table 2:** Evaluation of the decadal contributions of land use and climate change to the local hydrology by comparison of the differences of simulated water yield between two scenarios in Heihe River Basin (HRB) with that for the three sub-basins.

Impact	Land use change	Climate change	Land use &climate change
1980s	M3–M1	M2–M1	M4–M1
1990s	M6–M4	M5–M4	M7–M4
2000s	M10–M8	M11–M10	M11–M8
1980–2009	M3 + M6 + M10−M1−M4−M8	M2 + M5 + M11−M1−M4−M10	M4 + M7 + M11−M1−M4−M8

**Table 3:** Details of the computations used to determine the impact of land use and climate change on the changes in hydrological process in the 1980s in Heihe River Basin (HRB) and its sub-basin in Northwest China.

Variable	M1	M2	M3	M4	Residual
Water Yield (mm)	Y_M1_	Y_M2_	Y_M3_	Y_M4_	
Modeling change (mm)		Y_M2_–Y_M1_	Y_M3_–Y_M1_	Y_M4_–Y_M1_	(Y_M4_–Y_M1_) − (Y_M2_–Y_M1_) − (Y_M3_–Y_M1_)
Contribution rate (%)		*CR*_land 80s_	*CR*_climate 80s_	100%	*CR*_residual_

## References

[b1] Piao S. L. . The impacts of climate change on water resources and agriculture in China. Nature 467, 43–51, doi: 10.1038/nature09364 (2010). 20811450

[b2] Notter B., Hurni H., Wiesmann U. & Abbaspour K. C. Modelling water provision as an ecosystem service in a large East African river basin. Hydrol Earth Syst Sc 16, 69–86, doi: 10.5194/hess-16-69-2012 (2012).

[b3] Cheng Y.-f., Wang G.-x. & Xi H.-y. & Wang, J.-d. Variations of land evapotranspiration in the plain of the middle reaches of Heihe River in the recent 35 years. Journal of Glaciology and Geocryology 29, 406–412 (2007).

[b4] Jeuland M. & Whittington D. Water resources planning under climate change: Assessing the robustness of real options for the Blue Nile. Water Resour Res 50, 2086–2107, doi: 10.1002/2013wr013705 (2014).

[b5] Zhang Y. G. . Impact of projected climate change on the hydrology in the headwaters of the Yellow River basin. Hydrol Process 29, 4379–4397, doi: 10.1002/hyp.10497 (2015).

[b6] Zuo D. P. . Simulating spatiotemporal variability of blue and green water resources availability with uncertainty analysis. Hydrol Process 29, 1942–1955, doi: 10.1002/hyp.10307 (2015).

[b7] Qiao L. . Climate Change and Hydrological Response in the Trans-State Oologah Lake Watershed-Evaluating Dynamically Downscaled NARCCAP and Statistically Downscaled CMIP3 Simulations with VIC Model. Water Resour Manag 28, 3291–3305, doi: 10.1007/s11269-014-0678-z (2014).

[b8] Ravazzani G. . Investigation of Climate Change Impact on Water Resources for an Alpine Basin in Northern Italy: Implications for Evapotranspiration Modeling Complexity. Plos One 9, doi: ARTN e10905310.1371/journal.pone.0109053 (2014). 10.1371/journal.pone.0109053 PMC4186761 25285917

[b9] Kundzewicz Z. W. . The implications of projected climate change for freshwater resources and their management. Hydrolog Sci J 53, 3–10 (2008).

[b10] Lee K. S. & Chung E. S. Hydrological effects of climate change, groundwater withdrawal, and land use in a small Korean watershed. Hydrol Process 21, 3046–3056 (2007).

[b11] Abbaspour K. C., Faramarzi M., Ghasemi S. S. & Yang H. Assessing the impact of climate change on water resources in Iran. Water Resour Res 45 (2009).

[b12] Bekele E. G. & Knapp H. V. Watershed Modeling to Assessing Impacts of Potential Climate Change on Water Supply Availability. Water Resour Manag 24, 3299–3320, doi: 10.1007/s11269-010-9607-y (2010).

[b13] Wang Z. G., Ficklin D. L., Zhang Y. Y. & Zhang M. H. Impact of climate change on streamflow in the arid Shiyang River Basin of northwest China. Hydrol Process 26, 2733–2744, doi: 10.1002/hyp.8378 (2012).

[b14] Huo A. D. & Li H. Assessment of climate change impact on the stream-flow in a typical debris flow watershed of Jianzhuangcuan catchment in Shaanxi Province, China. Environmental Earth Sciences 69, 1931–1938, doi: 10.1007/s12665-012-2025-0 (2013).

[b15] Lee A., Cho S., Kang D. K. & Kim S. Analysis of the effect of climate change on the Nakdong river stream flow using indicators of hydrological alteration. J Hydro-Environ Res 8, 234–247, doi: 10.1016/j.jher.2013.09.003 (2014).

[b16] Murphy K. W. & Ellis A. W. An assessment of the stationarity of climate and stream flow in watersheds of the Colorado River Basin. Journal of Hydrology 509, 454–473, doi: 10.1016/j.jhydrol.2013.11.056 (2014).

[b17] Geriesh M. H., Balke K. D., El-Rayes A. E. & Mansour B. M. Implications of climate change on the groundwater flow regime and geochemistry of the Nile Delta, Egypt. J Coast Conserv 19, 589–608, doi: 10.1007/s11852-015-0409-5 (2015).

[b18] Goodarzi M., Abedi-Koupai J., Heidarpour M. & Safavi H. R. Evaluation of the Effects of Climate Change on Groundwater Recharge Using a Hybrid Method. Water Resour Manag 30, 133–148, doi: 10.1007/s11269-015-1150-4 (2016).

[b19] Maxwell R. M. . The imprint of climate and geology on the residence times of groundwater. Geophysical Research Letters 43, 701–708, doi: 10.1002/2015gl066916 (2016).

[b20] Meixner T. . Implications of projected climate change for groundwater recharge in the western United States. Journal of Hydrology 534, 124–138, doi: 10.1016/j.jhydrol.2015.12.027 (2016).

[b21] Dams J., Nossent J., Senbeta T. B., Willems P. & Batelaan O. Multi-model approach to assess the impact of climate change on runoff. Journal of Hydrology 529, 1601–1616, doi: 10.1016/j.jhydrol.2015.08.023 (2015).

[b22] Kabiri R., Bai V. R. & Chan A. Assessment of hydrologic impacts of climate change on the runoff trend in Klang Watershed, Malaysia. Environmental Earth Sciences 73, 27–37, doi: 10.1007/s12665-014-3392-5 (2015).

[b23] Chang H. J. & Jung I. W. Spatial and temporal changes in runoff caused by climate change in a complex large river basin in Oregon. Journal of Hydrology 388, 186–207, doi: 10.1016/j.jhydrol.2010.04.040 (2010).

[b24] Chen Y. N., Takeuchi K., Xu C. C., Chen Y. P. & Xu Z. X. Regional climate change and its effects on river runoff in the Tarim Basin, China. Hydrol Process 20, 2207–2216, doi: 10.1002/hyp.6200 (2006).

[b25] Liu M. L., Adam J. C. & Hamlet A. F. Spatial-temporal variations of evapotranspiration and runoff/precipitation ratios responding to the changing climate in the Pacific Northwest during 1921-2006. J Geophys Res-Atmos 118, 380–394, doi: 10.1029/2012jd018400 (2013).

[b26] Liu Y. L. . Response of evapotranspiration and water availability to changing climate and land cover on the Mongolian Plateau during the 21st century. Global Planet Change 108, 85–99, doi: 10.1016/j.gloplacha.2013.06.008 (2013).

[b27] Liu Y. L. . Response of evapotranspiration and water availability to the changing climate in Northern Eurasia. Climatic Change 126, 413–427, doi: 10.1007/s10584-014-1234-9 (2014).

[b28] Pan S. F. . Responses of global terrestrial evapotranspiration to climate change and increasing atmospheric CO2 in the 21st century. Earths Future 3, 15–35, doi: 10.1002/2014ef000263 (2015).

[b29] Algeet-Abarquero N., Marchamalo M., Bonatti J., Fernandez-Moya J. & Moussa R. Implications of land use change on runoff generation at the plot scale in the humid tropics of Costa Rica. Catena 135, 263–270, doi: 10.1016/j.catena.2015.08.004 (2015).

[b30] Rodriguez-Lloveras X. . Patterns of runoff and sediment production in response to land-use changes in an ungauged Mediterranean catchment. Journal of Hydrology 531, 1054–1066, doi: 10.1016/j.jhydrol.2015.11.014 (2015).

[b31] Sajikumar N. & Remya R. S. Impact of land cover and land use change on runoff characteristics. J Environ Manage 161, 460–468, doi: 10.1016/j.jenvman.2014.12.041 (2015). 25575849

[b32] Ghimire C. P., Bruijnzeel L. A., Lubczynski M. W. & Bonell M. Negative trade-off between changes in vegetation water use and infiltration recovery after reforesting degraded pasture land in the Nepalese Lesser Himalaya. Hydrol Earth Syst Sc 18, 4933–4949, doi: 10.5194/hess-18-4933-2014 (2014).

[b33] Neris J., Jimenez C., Fuentes J., Morillas G. & Tejedor M. Vegetation and land-use effects on soil properties and water infiltration of Andisols in Tenerife (Canary Islands, Spain). Catena 98, 55–62, doi: 10.1016/j.catena.2012.06.006 (2012).

[b34] Deng X. Z., Shi Q. L., Zhang Q., Shi C. C. & Yin F. Impacts of land use and land cover changes on surface energy and water balance in the Heihe River Basin of China, 2000–2010. Physics and Chemistry of the Earth 79– 82, 2–10, doi: 10.1016/j.pce.2015.01.002 (2015).

[b35] Metzger J. C., Landschreiber L., Grongroft A. & Eschenbach A. Soil evaporation under different types of land use in southern African savanna ecosystems. J Plant Nutr Soil Sc 177, 468–475, doi: 10.1002/jpln.201300257 (2014).

[b36] House-Peters L. A. & Chang H. Modeling the impact of land use and climate change on neighborhood-scale evaporation and nighttime cooling: A surface energy balance approach. Landscape Urban Plan 103, 139–155, doi: 10.1016/j.landurbplan.2011.07.005 (2011).

[b37] Cheng G. D. . Integrated study of the water-ecosystem-economy in the Heihe River Basin. Natl Sci Rev 1, 413–428, doi: 10.1093/nsr/nwu017 (2014).

[b38] Hu X. L., Lu L., Li X., Wang J. H. & Guo M. Land Use/Cover Change in the Middle Reaches of the Heihe River Basin over 2000-2011 and Its Implications for Sustainable Water Resource Management. Plos One 10, doi: ARTN e012896010.1371/journal.pone.0128960 (2015). 10.1371/journal.pone.0128960 PMC4482701 26115484

[b39] Kueppers L. M. & Snyder M. A. Influence of irrigated agriculture on diurnal surface energy and water fluxes, surface climate, and atmospheric circulation in California. Clim Dynam 38, 1017–1029, doi: 10.1007/s00382-011-1123-0 (2012).

[b40] Chen Y. . Water demand management: A case study of the Heihe River Basin in China. Physics and Chemistry of the Earth 30, 408–419, doi: DOI 10.1016/j.pce.2005.06.019 (2005).

[b41] Zhou G. Y., Wei X. H. & Yan J. H. Impacts of eucalyptus (Eucalyptus exserta) plantation on sediment yield in Guangdong Province, Southern China - a kinetic energy approach. Catena 49, 231–251, doi: 10.1016/S0341-8162(02)00030-9 (2002).

[b42] Zhou G. . Global pattern for the effect of climate and land cover on water yield. Nature Communications 6, 5918, doi: 10.1038/ncomms6918 (2015). 25574930

[b43] Wang P., Yu J. J., Pozdniakov S. P., Grinevsky S. O. & Liu C. M. Shallow groundwater dynamics and its driving forces in extremely arid areas: a case study of the lower Heihe River in northwestern China. Hydrol Process 28, 1539–1553, doi: 10.1002/hyp.9682 (2014).

[b44] Jian Z. . Improveed SEBS model for evaluation irrigation water use efficiency in the middle reaches of Heihe River. J Hydraul Eng 45, 1387–1394, doi: 10.13243/j.cnki.slxb.2014.12.001 (2014).

[b45] Wei L., Tao W., Hang Z. & Zhenzhen M. Drivers forces of different type of land desertification in Heihe River Basin. Journal of Desert Research 28, 634–640 (2008).

[b46] Jun M. . Characteristics and influencing facyors of land desertification in Kern Oasis area of low Heihe Basin in recent 20 years. Bulletin of Soil and Water Conservation 34, 161–165, doi: 10.13961/j.cnki.stbctb.2014.01.025 (2014).

[b47] Fu B., Wenpeng L. & Zhiheng L. analysis on the main causes resulting in vegetation degeneration in the Heihe River Basin. Arid Zone Research 25, 220–224, doi: 10.13866/j.azr.2008.02.014 (2008).

[b48] IPCC. Climate Change 2013:The Physical Science Basis. (Cambridge University Press, 2007).

[b49] Tao F. & Zhang Z. Adaptation of maize production to climate change in North China Plain: Quantify the relative contributions of adaptation options. European Journal of Agronomy 33, 103–116, doi: 10.1016/j.eja.2010.04.002 (2010).

[b50] Su T., Feng T. C. & Feng G. L. Evaporation variability under climate warming in five reanalyses and its association with pan evaporation over China. J Geophys Res-Atmos 120, 8080–8098, doi: 10.1002/2014jd023040 (2015).

[b51] Abouabdillah A. . Evaluation of soil and water conservation measures in a semi-arid river basin in Tunisia using SWAT. Soil Use Manage 30, 539–549, doi: 10.1111/sum.12146 (2014).

[b52] Li D. L., Feng J. Y., Chen L., Liu H. L. & Zhang J. Study on interdecadal change of Heihe runoff and Qilian mountain’s climate. Plateau Meteorol 22, 104–110 (2003).

[b53] Wu W. Z. . Multi-scale analysis of the long-term trend of the hydromeorological variablesin of the upper reach of the Heihe River,Northwest China. Marine Geology and Quaternary Geology 33, 37–44 (2013).

[b54] Haijun W. . The spatial-temporal climate and the response of runoff in the past 48a of the Zhangye region in the middle reaches of the Hehe River. Journal of Arid Land Resources and Environment 24, 82–88, doi: 10.13448/j.cnki.jalre.2010.02.009 (2010).

[b55] Jun W. & Jijun M. Characteristics and tendencies of annual runoff variations in the Heihe River Basin during the past 60 years. Scienrtia Geographica Sinica 28, 83–88, doi: 10.13249/j.cnki.sgs.2008.01.005 (2008).

[b56] Li Z. H., Deng X. Z., Wu F. & Hasan S. S. Scenario Analysis for Water Resources in Response to Land Use Change in the Middle and Upper Reaches of the Heihe River Basin. Sustainability 7, 3086–3108, doi: DOI 10.3390/su7033086 (2015).

[b57] Zhang L., Nan Z. T., Yu W. J. & Ge Y. C. Modeling Land-Use and Land-Cover Change and Hydrological Responses under Consistent Climate Change Scenarios in the Heihe River Basin, China. Water Resour Manag 29, 4701–4717, doi: 10.1007/s11269-015-1085-9 (2015).

[b58] Wu Y. P. . Diagnosing Climate Change and Hydrological Responses in the Past Decades for a Minimally-disturbed Headwater Basin in South China. Water Resour Manag 28, 4385–4400, doi: 10.1007/s11269-014-0758-0 (2014).

[b59] Awan U. K. & Ismaeel A. A new technique to map groundwater recharge in irrigated areas using a SWAT model under changing climate. Journal of Hydrology 519, 1368–1382, doi: 10.1016/j.jhydrol.2014.08.049 (2014).

[b60] Moriasi D. N. . Model evaluation guidelines for systematic quantification of accuracy in watershed simulations. T Asabe 50, 885–900 (2007).

[b61] Troin M. & Caya D. Evaluating the SWAT’s snow hydrology over a Northern Quebec watershed. Hydrol Process 28, 1858–1873, doi: 10.1002/hyp.9730 (2014).

[b62] Ahl R. S., Woods S. W. & Zuuring H. R. Hydrologic Calibration and Validation of SWAT in a Snow-Dominated Rocky Mountain Watershed, Montana, USA. J Am Water Resour As 44, 1411–1430, doi: 10.1111/j.1752-1688.2008.00233.x (2008).

