# Supplementary material for: Attribution of hydrological change in Heihe River Basin to climate and land use change in the past three decades
Source: Sci Rep. 2016 Sep 20;6:33704. doi: 10.1038/srep33704 (PMC5028708; doi:10.1038/srep33704)
Supplement: Supplementary Information [file srep33704-s1.pdf]

Attribution of hydrological change in the Heihe River Basin to climate and land use change in the past three decades

Kaisheng Luo[1,2], Fulu Tao[1,]* , Juana P. Moiwo[3] Dengpan, Xiao[4]

[1]Key Laboratory of Land Surface Pattern and Simulation, Institute of Geographical Sciences and Natural Resources Research, Chinese Academy of Sciences, Beijing 100101, China

[2]University of Chinese Academy of Sciences, Beijing 100101, China

[3] Department of Agricultural Engineering, School of Technology, Njala University, Sierra Leone

[4]Institute of Geographical Sciences, Hebei Academy of Sciences, Shijiazhuang 050011, China

The supplementary material file includes:

Supplementary Figures S1-S3

Supplementary Tables S1-S3

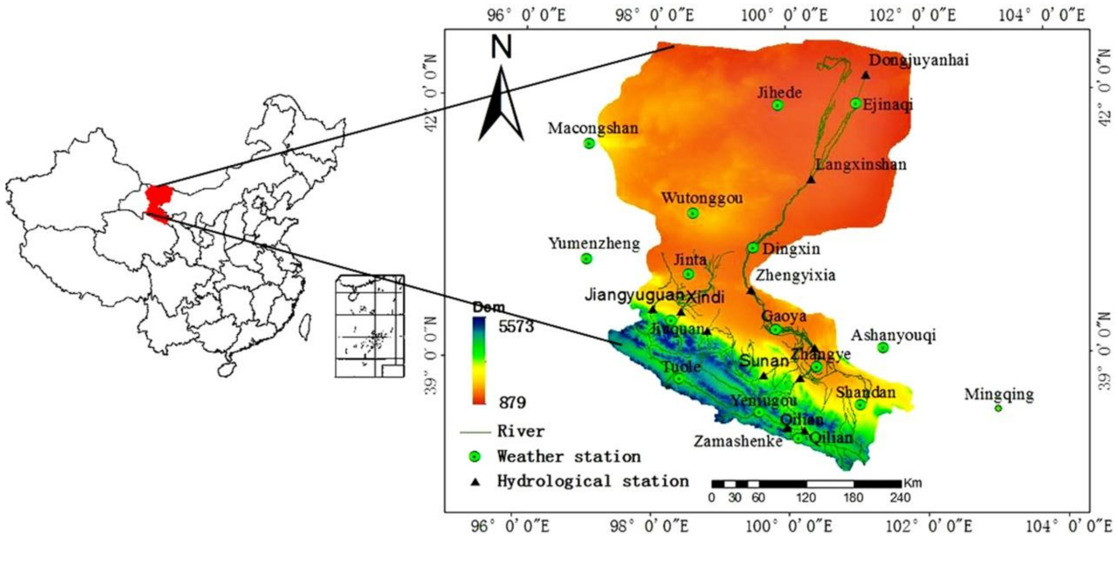

Figure S1. A map depicting the location of Heihe River Basin in China (left plate) and the expanded study area (right plate) depicting the elevation, river and data stations used in the study. This figure was made in the ArcGIS 10.2 software provided by Environmental Systems Research Institute (http://www.esri.com/)

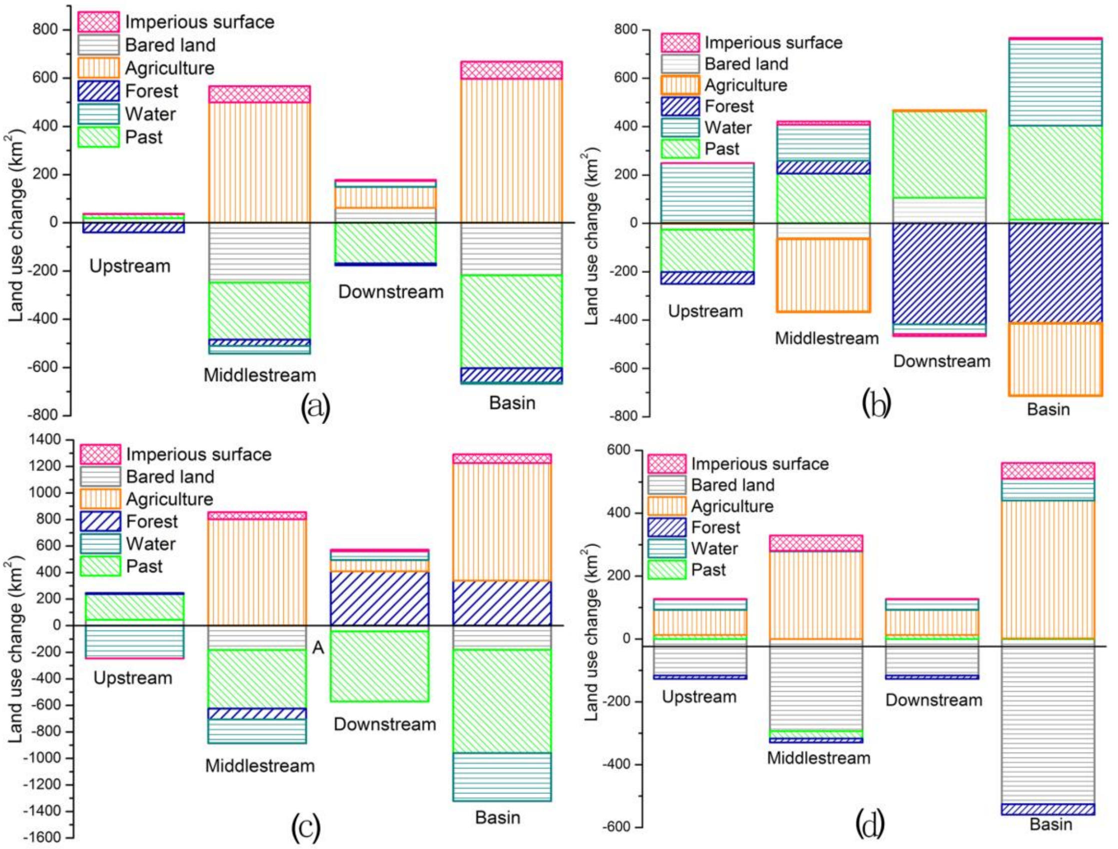

Figure S2. Plots of land use change in Heihe River Basin in Northwest China in the last three decades: (a) Land use change in 1980–2010, which is the difference between land use in 1985 and that in 2008;. (b) Land use change for the period from the 1980s to 1990s, which is the difference between land use in 1985 and that in 1995; (c) Land use change for the period from the 1990s to 2000s, which is the difference between land use in 1995 and that in 2005; and (d) Land use change for the period from the 2000s to 2010s, which is the difference between land use in 2000 and that in 2008.

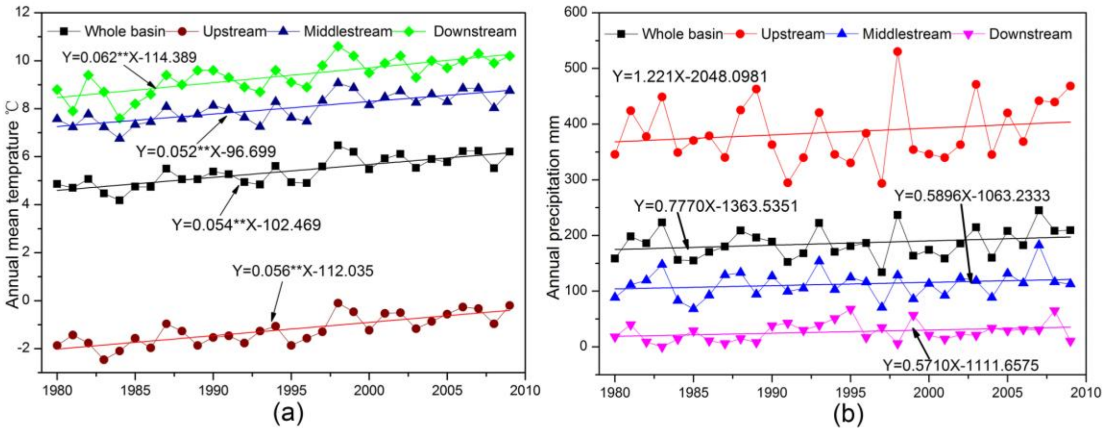

Figure S3. Trends in annual mean temperature (a) and annual precipitation (b) for 1980–2009. Note that trends with single asterisks (*) are significant at the 5% probability level and the ones with double asterisks (**) are significant at the 1% probability level.

Table S1. Performance of the Soil and Water Assessment Tool (SWAT) used in the calibration and validation analyses at the yearly scale.

Station	Calibration analysis				Validation analysis			
	R^2	*NS*	*RSR*	*PBIAS (%)*	R^2	*NS*	*RSR*	*PBIAS (%)*
Qilian	0.80	0.75	0.28	6	0.88	0.70	0.81	5
Zamashenke	0.92	0.78	0.23	4	0.90	0.74	0.80	6
Sunan	0.90	0.78	0.24	6	0.82	0.72	0.79	8
Fenglehe	0.90	0.76	0.26	5	0.81	0.74	0.79	7
Xindi	0.92	0.79	0.23	7	0.88	0.72	0.80	8
Jiayuguan	0.69	0.66	0.32	11	0.66	0.43	0.72	10
Yinluoxia	0.85	0.75	0.28	9	0.80	0.73	0.74	11
Gaoya	0.65	0.68	0.41	12	0.63	0.50	0.64	11
Zhengyixia	0.68	0.67	0.47	12	0.63	0.55	0.65	13
Langxinshan	0.53	0.66	0.51	13	0.50	0.40	0.61	14
Dongjuyanhai	0.51	0.65	0.53	14	0.50	0.41	0.61	14

Table S2. Performance of the Soil and Water Assessment Tool (SWAT) in the calibration and validation analyses at the monthly scale.

Station	Calibration analysis				Validation analysis			
	R^2	*NS*	*RSR*	*PBIAS (%)*	R^2	*NS*	*RSR*	*PBIAS (%)*
Qilian	0.94	0.72	0.31	8	0.88	0.70	0.35	9
Zamashenke	0.95	0.76	0.28	7	0.90	0.74	0.28	10
Sunan	0.96	0.76	0.30	9	0.82	0.72	0.22	10
Fenglehe	0.93	0.74	0.32	11	0.81	0.74	0.32	12
Xindi	0.95	0.76	0.28	7	0.88	0.72	0.31	9
Jiayuguan	0.76	0.56	0.47	14	0.66	0.43	0.41	16
Yinluoxia	0.90	0.71	0.36	12	0.80	0.73	0.39	14
Gaoya	0.78	0.57	0.46	18	0.63	0.50	0.46	21
Zhengyixia	0.73	0.56	0.49	18	0.63	0.55	0.52	21
Langxinshan	0.64	0.41	0.64	21	0.50	0.40	0.65	22
Dongjuyanhai	0.63	0.42	0.66	22	0.50	0.41	0.65	22

Table S3.Comparison between simulated and observed water yield per decade

Scenario	Land use	Climate	Observed (mm)	Simulated (mm)	Relative error (%)
M1	1980s	1980s	206.12	200.72	2.62
M4	1990s	1990s	214.55	210.22	2.02
M7	2000s	2000s	231.42	223.49	3.42
